# Development and Validation of a Questionnaire Evaluating the Impact of Prosthetic Dental Treatments on Patients’ Oral Health Quality of Life: A Prospective Pilot Study

**DOI:** 10.3390/ijerph17145037

**Published:** 2020-07-13

**Authors:** Eitan Mijiritsky, Yael Lerman, Ori Mijiritsky, Asaf Shely, Joseph Meyerson, Maayan Shacham

**Affiliations:** 1 Department of Otolaryngology, Head and Neck and Maxillofacial Surgery, Tel-Aviv Sourasky Medical Center, Sackler Faculty of Medicine, Tel Aviv 6139001, Israel; mijiritsky@bezeqint.net; 2 The Maurice and Gabriela Goldschleger School of Dental Medicine, Tel Aviv University, Tel Aviv 6997801, Israel; 3 Private Practice, Tel Aviv 6971915, Israel; yaelerman@gmail.com; 4 Department of Psychology, Tel Aviv-Yafo Academic College, Tel Aviv 6818543, Israel; orimijiritsky2@gmail.com; 5 Department of Prosthodontics, The Maurice and Gabriela Goldschleger School of Dental Medicine, Tel Aviv University, Tel Aviv 6997801, Israel; 6 Department of Psychology, Bar Ilan University, Ramat Gan 5290002, Israel; hypnoclinic10@gmail.com; 7 School of Social Work, Ariel University, Ariel 40700, Israel; Drmaayanshacham@gmail.com

**Keywords:** oral health related quality of life (OHRQoL), psychological impacts, prosthodontics, esthetics

## Abstract

Objectives: the aims of this study were the development of a novel questionnaire to assess the impact of prosthetic treatments on oral health-related quality of life (OHRQoL) and the performance of a prospective pilot study. Background: the currently preferred OHRQoL measurement tool is the oral health impact profile-49 (OHIP-49), a self-report questionnaire which mainly focuses on general effects related to oral health. Materials and methods: A total of 24 adult participants (9 females and 15 males) were recruited and asked to complete the novel questionnaire twice: once before the prosthetic treatment began and 4–6 weeks post-treatment. The assessment of the change in OHRQoL was based on the differences in participants’ answers before and after treatment. Data were analyzed using ANOVA with a repeated-measures method and *t*-tests. The reliability of the questionnaire was tested using Cronbach’s alpha and intraclass coefficient (ICC). Results: The questionnaire was found to be reliable (α ≥ 0.6), with “social disability” having the highest score (α = 0.868). All domains showed an improvement (α < 0.005) in OHRQoL scores after treatment. Conclusions: the novel questionnaire tested in this study was found to be reliable and convenient to use, and demonstrated that prosthetic treatments have a significant positive effect on OHRQoL post-treatment scores.

## 1. Introduction

Dental and oral health both seem to have a significant impact on self-perception, psychological status, and human social status [1,2]. These factors affect self-esteem and the individual’s overall quality of life (QoL) [1,2,3]. Patients’ expectations of good oral health include a number of specific characteristics related but not limited to function, comfort, and appearance [4,5]. Dental aesthetics is a key factor in determining an individual’s attractiveness. Overall, physical attractiveness in general and facial attractiveness in particular have a huge role to play in social interaction. It affects friendships, personal assessments, performance, success in finding a partner, and even the chances of getting a job [2,6]. Several studies had reported a correlation between self-report of attractiveness and traits such as dominance, mental stability, and anxiety [2,6]. When dental health is impaired due to tooth loss or any other reason, one’s function, comfort, and appearance may be impaired [2,4,7,8]. Consequently, such impairment may have a detrimental effect on psychological and social measures, such as self-confidence, social avoidance, anxiety, and emotional distress [1,5].

Oral health related quality of life (OHRQoL) is a broad term which deals with such psychological, social and emotional aspects of oral health [5,8], and its effect on one’s everyday life [4,9]. The most commonly used tool to assess OHRQoL is the oral health impact profile-49 (OHIP-49). This questionnaire contains 49 questions which are based on the theoretical model developed by the World Health Organization (WHO) and adjusted to the measures of oral health by Locker [5,10,11]. It is a self-report questionnaire which assesses any dysfunction, discomfort, and/or other limitations due to different oral conditions [5]. It deals with three domains—social, psychological, and physiological—and has several sub-scales including functional limitation, physical pain, psychological discomfort, physical disability, psychological disability, social disability, and handicap [12]. The questionnaire also has a short version, known as the oral health impact profile-14 (OHIP-14) [13]. However, these self-report tools have certain limitations, such as, they do not report the level of satisfaction with one’s oral health [5].

Dental prosthetic treatments may help in dealing with concerns regarding lack of aesthetics or dysfunctions, as most patients report high levels of satisfaction and increase in OHRQoL, as demonstrated in various studies [14,15,16,17,18,19]. Despite this, both OHIP-49 and OHIP-19 are not used in routine dental care, as they do not relate to the effects exerted by the dental practitioner on the success of the prosthetic treatment [1,20].

In light of the above, there seems to be a need to evaluate a tool that can used in routine dental care and assess the psychosocial effects of dental prosthetic treatments. Such a tool may help dental practitioners to better evaluate the importance of mental and social aspects of dental prosthetic treatments and their effects on their patients. In turn, it may lead to a better communication between the patient and the dental practitioner and ultimately lead to a better treatment outcome [1,4,20]. Therefore, the objective of the current study was the development of a novel questionnaire and to examine its ability to evaluate OHRQoL before and after prosthetic treatments.

## 2. Materials and Methods

### 2.1. Novel Questionnaire Development

#### Literature Review and Meta-Synthesis

The novel questionnaire was developed based on a systemic literature review and meta-analysis on articles published in Medline between the years 1985–2016 and included two search engines—PubMed and Ebsco—with initial filtering of the system by language (English), followed by a manual search using the keywords: psychological impacts, prosthodontics, oral health-related quality of life (OHRQoL), aesthetics, and combinations of the abovementioned.

The literature lists were reviewed in order to determine which articles have the potential to identify metrics for the psychosocial effects on dental rehabilitation outcomes. The potential articles were those that reviewed and/or provided information about the indicators relevant to the effects we have defined, in the context of two main treatment modalities: removable versus fixed rehabilitations. Based on the conclusions that emerged from the literature review and the meta-synthesis process, a new questionnaire based on the relevant psychological metrics was formulated. The questionnaire is based largely on questions from the OHIP-49 and PIDAQ questionnaires. The novel questionnaire was developed by three authors: an oral rehabilitation specialist (EM), a clinical psychologist (JM), and a dental student (YL). The guidelines for writing the questionnaire were: contain fewer questions than OHIP-49 in order to be more convenient to use, rephrasing of questions whose answers were restrictive so that more patients will be able to agree with the saying, and removal of questions which were too specific, such as “have you had sore jaw” and “have you had painful gums”; the latter of these was replaced by a single question: “Do you have any pain in your mouth?” In addition, questions that were similar in meaning were deleted (for example, the two questions “Has your speech been unclear” and “Have people misunderstood some of your words” were compiled to a single item “Have you had trouble pronouncing words?”).

The questionnaire contains 31 questions and statements, which are divided into six dimensions: functional disability, physiological pain, psychological discomfort, physiological disability, psychological disability, and social disability (Table 1). The answers to the questions are on a Likert scale of 1–5, which refers to the prevalence of the phenomenon occurring in the section during a pre-defined period (last month), so that 5 = very often and 1 = never. Each question also has a neutral answer that means irrelevant/unknowable. 

The final questionnaire is attached to this work as Appendix A, while the source questionnaires are Appendix B and Appendix C, respectively.

After approval of the constructed questionnaire by the Tel-Aviv University Ethical Committee, a preliminary research was conducted to test the research hypothesis.

### 2.2. PICO Defined for the Current Study

#### 2.2.1. The Study Population

The sample included 24 Israeli adults (9 female and 15 male, mean age 51.13 years) who were treated by students at the Tel Aviv University School of Dentistry and underwent a dental rehabilitation process (lasting between 6–26 months), due to complete/partial tooth loss or teeth discoloration. 

Inclusion criteria:

- Over 18 years of age and under 75 years.
- Patients at the School of Dentistry at Tel Aviv University.
- Good general health, no medical risks, including past or present chemotherapy or radiation.
- Ability to understand, respond, and complete the questionnaire.
- At least one tooth in need of prosthetic treatment: fixed partial denture (FPD), implant-supported, full or partial removable restorations, full or partial removable restorations supported by implants.

**Exclusion criteria:**

- Systemic illnesses that will prevent research participation.
- Oral disease that directly or indirectly affects rehabilitation.
- Oral disease that directly or indirectly affects OHRQoL.
- Failure to complete prosthetic treatments.

#### 2.2.2. Intervention

Patients were asked to complete the new questionnaire twice: once prior to the onset of rehabilitation and approximately 6 weeks after the treatments’ completion. The internal consistency of the questionnaire before and after prosthetic treatments was statistically examined, being divided into the sub-topics, according to Cronbach’s alpha (α ≥ 0.6). The reliability of the different domains was calculated and presented as intraclass coefficient (ICC) [21].

#### 2.2.3. Outcome

Assessment of the psychological implications and improvement in the patients’ QoL indices were made based on the answers given to the same question before and after treatment and were measured in specific patient-based measurements, which examined (for each of the questionnaires) differences between before and after prosthetic treatment as well as differences between the different types of treatment.

##### Documentation and information collection

The questionnaires and informed consent forms were provided directly to the patients during their visit to the university student clinics or in a personal meeting with them at the end of the rehabilitation and were manually filled.

The gathered information was stored in an Excel file; data was stored in an examiner computer (Y.L) without enclosing any identifying details of the participants. The data included serial number of the respondent, age, gender, type of treatment, duration of treatment, and answers to the different items of the novel questionnaire. 

##### Statistical analysis

Statistical analysis was performed using the Statistical Package for Social Sciences for Windows Release 23.0 (SPSS Inc., Chicago, IL, USA).

The answers given to the questionnaires were analyzed using ANOVA with repeated measures analysis in order to evaluate the differences between before and after treatment, as well as differences between the permanent and removable rehabilitation groups. T-test and variance analysis were used to examine the relationships between subjects’ characteristics (gender, age, duration of treatment, etc.) and their effects on patients responses to the questionnaire.

## 3. Results

### 3.1. Questionnaire Reliability

The internal consistency for the different questionnaire domains was found to be above 0.6, as detailed in Table 2. The ICC for each domain was calculated and was found to be moderate for most domains (0.5 < x < 0.75) and good for the social disability domain (0.75 < x < 0.9).

### 3.2. Type of Rehabilitation

Most treatments appeared to be under the subsection of fixed rehabilitation (16 patients) versus removable rehabilitation (8 patients). Under the removable rehabilitation sub-subject: full and partial removable dentures were included, and under the fixed rehabilitation sub-subject: crowns and bridges, implant supported crowns and bridges, implant supported fixed partial dentures, and implant supported partial dentures.

The changes in OHRQoL values before and after treatment, combined with the effect of the type of prosthetic rehabilitation on this change, were subdivided into the sub-themes in the questionnaire and tested using ANOVA with repeated measures.

An improvement in OHRQoL was achieved for each of the indices at the end of treatment. 

### 3.3. Functional Disability

**Figure 1:**
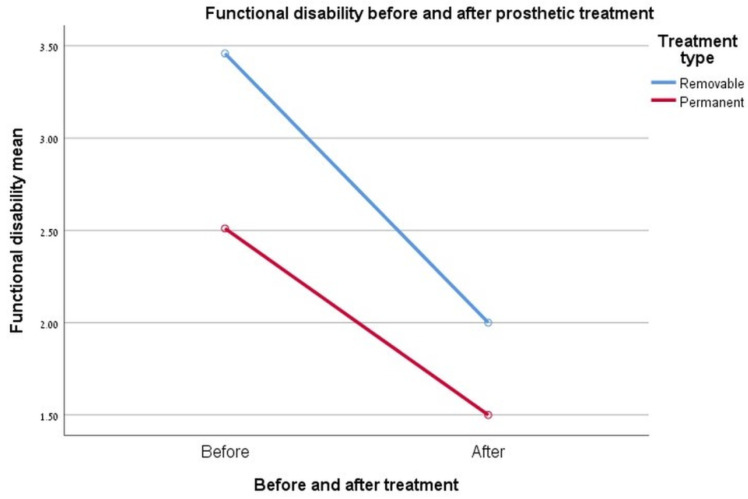
Graph showing the change in the “functional disability” index.

The degree of satisfaction (= removal of the functional limit) obtained at the end of rehabilitation was significantly higher than that measured before the start of treatment for both types of rehabilitations (D < 0.005). The two-factor ANOVA did not demonstrate a significant difference in the relationship between the type of rehabilitation and the degree of improvement in patient satisfaction at the end of rehabilitation. See Figure 1.

### 3.4. Physiological Pain

**Figure 2:**
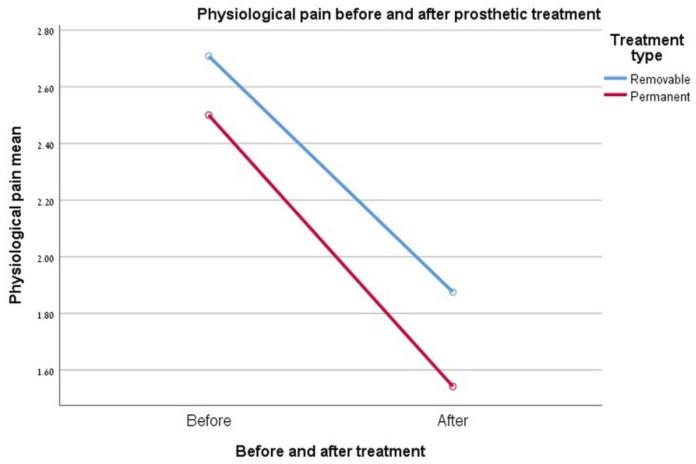
Graph showing the change in the “physiological pain” index.

The reported pain at the end of rehabilitation was significantly lower than that measured before the start of treatment for both types of rehabilitation (D < 0.005). The two-way ANOVA did not show a significant difference in the relationship between the type of rehabilitation and the degree of pain reduction of the patient at the end of rehabilitation. Moreover, the sum of the scores obtained for each type of rehabilitation (before and after rehabilitation) was very similar for both groups, meaning the degree of pain and the degree of pain improvement is very similar between the two groups at the two time points examined. See Figure 2.

### 3.5. Psychological Discomfort

**Figure 3:**
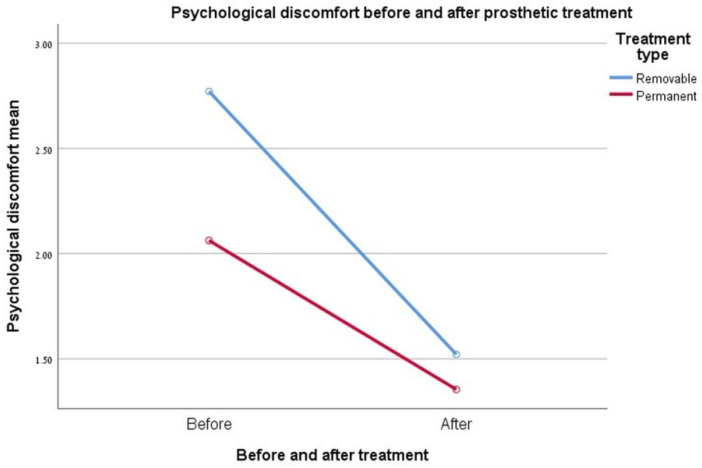
Graph showing the change in the “psychological discomfort” index.

The difference between rehabilitation types and their effect on the change in the psychological discomfort index was found to be insignificant (0.061, *p* ≤ 0.05). It can be seen that removable rehabilitation group) showed higher pre-treatment values. However, the degree of improvement for this measure at the end of rehabilitation appears to have been slightly more in the removable rehabilitation group than the degree of improvement in the permanent rehabilitation group. See Figure 3.

### 3.6. Physiological Disability

**Figure 4:**
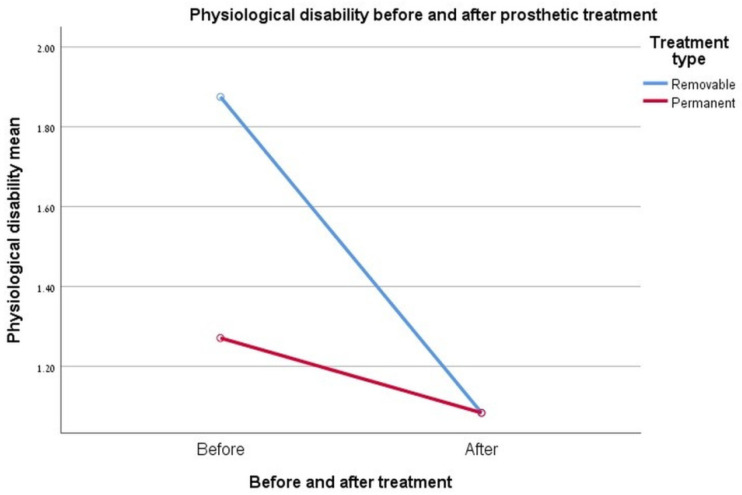
Graph showing the change in the “physiological disability” index.

Interaction results (0.084, *p* ≤ 0.05) between treatment type and its effect on the degree of improvement in the physiological disability index were not significant. As can be seen in Figure 4, a significant change in values was achieved at the end of treatment with the removable rehabilitation group, compared to before prosthetic treatments conductance. Because the permanent rehabilitation group presented very low values in the pre-treatment questionnaire (no physiological limitation is reported), there was actually little room for improvement in the index in this group. At the end of the treatment, both groups presented an almost identical score, that is, a significant improvement and the removal of the limit for the removable rehabilitation group. See Figure 4.

### 3.7. Psychological Disability

**Figure 5:**
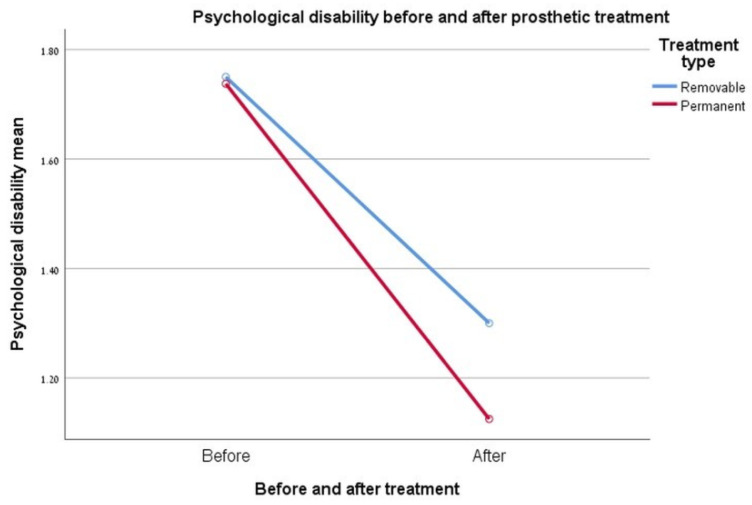
Graph showing the change in the “psychological disability” index.

A significant difference in the improvement was observed before the start of rehabilitation versus the values at the end, for each type of rehabilitation. In contrast, the relationship between the type of treatment and its effect on the degree of improvement in the psychological discomfort index was not found to be significant. In addition, the mean values obtained for this measure before the start of treatment were almost the same for both types of rehabilitation (removable 1.75 vs. constant 1.73), while the fixed rehabilitation group showed a marked improvement in the psychological limit at the end of treatment. See Figure 5.

### 3.8. Social Disability

**Figure 6:**
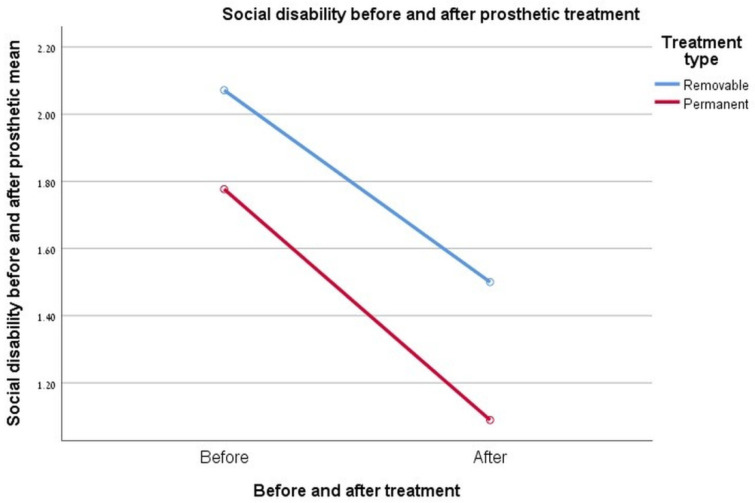
Graph showing the change in the “social disability” index.

Social limitation reporting values obtained at the end were significantly lower than those measured before treatment, for both types of rehabilitation (D < 0.005). The two-factor ANOVA did not demonstrate a significant difference in the relationship between the type of rehabilitation and the degree of improvement in the patient’s social disability at the end of rehabilitation. Moreover, the summary of the value gaps obtained for each type of rehabilitation (before and after rehabilitation) was very similar for both groups. Thus, the degree of improvement in this measure is very similar between the two groups at the two time points examined. See Figure 6.

### 3.9. The Physician

There was no correlation between the degree of satisfaction with the physician and the change in OHRQoL values at the end of treatment. This may be explained by the specific dental care given by students at the dental clinics at Tel Aviv University. The degree of satisfaction with the treatment was very high (an average score of 1.4 out of 5, with 1 being the maximum degree of satisfaction) for all patients.

### 3.10. Gender

A total of 15 male and 9 female adults participated in this study. The improvement in OHRQoL before and after rehabilitation was not certain on any of the measures in the questionnaire, except for the last measure: describing the degree of satisfaction with the physician (for which *p* = 0.035). The latter suggests that men exhibit a greater degree of satisfaction with the physician.

### 3.11. Rehabilitation Times

Dental rehabilitation times were divided into: up to 18 months of treatment (most of the study participants, approximately 17 patients), and over 18 months (7 patients). In analyzing the results obtained in relation to the relationship between duration of rehabilitation and degree of change in OHRQoL values, even in this case, t-test analysis did not show a significant relationship for any of the measures defined in the questionnaire, with the exception of the last measure, which describes the degree of satisfaction with the physician (*p* = 0.024). For this measure, the duration of treatment appeared to have a negative effect on the OHRQoL values tested in the questionnaire, so that patients whose treatment duration was shorter exhibited a significant improvement in the oral QoL values reported at the end of rehabilitation.

## 4. Discussion

There are relatively few studies dealing with the importance of psychosocial effects on the dental rehabilitation processes, along with the latter’s positive implications, such as improving QoL and sense of self-importance.

**Figure 7:**
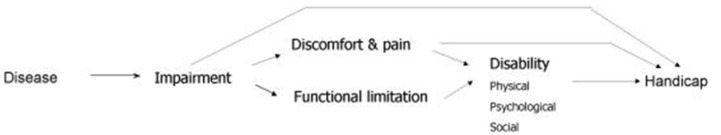
Conceptual model for measuring oral health.

Locker [10] formulated a model (see Figure 7) for measuring oral health—by expressing oral disease outcomes—from a biological and behavioral aspects of functional disability, discomfort, and dysfunction [4,10].

The model was pioneering in terms of the development of research in this field of dentistry. Beforehand, the psychosocial results of oral conditions have received little attention, as they are not considered to be life-threatening medical conditions. Furthermore, the oral cavity has been considered separate from the rest of the body throughout history, in relation to the person’s overall state of health [8,22].

Since being introduced in the 1990s, the OHIP-49 questionnaire has been translated into many languages, and despite its’ negative formulation, it is now one of the most instructive tools for assessing patient satisfaction with treatment [23]. Due to its reliability, we can compare the results of various studies on patients’ satisfaction with one treatment or another. This enables us to create dental guidelines for treatments. While using this questionnaire we can also compare various studies examining the social and psychological effects of oral health impairments [23,24].

Current studies suggest that tooth loss is associated not only with oral dysfunction, but also with loss of self-esteem and social status [11,14], while the restoration of the missing teeth and/or impaired denture brings with it a significant improvement in the OHRQoL values tested in comparison to pre-treatment [14,17,18,19]. This improvement was identified by the use of generic OHIP questionnaires, which examined the change in OHRQoL values as a research tool only, with the aim of examining the nature of one type of rehabilitation or the general benefits and effects of dental rehabilitation [22,25,26].

In the systematic review conducted as part of the current study, no studies were found to use OHIP questionnaires as part of routine clinical dental care. If to be used, such OHRQoL-related questionnaires may allow for a better understanding of the patient’s needs, or point to a need for adjustment of the type of dental treatment in order to better suit his or her initial psychosocial condition. To the best of our understanding, there is no questionnaire which can be used as a wide-ranging clinical tool, in addition to being a research tool, which focuses on such psychosocial influences and their implications on the individual’s oral QoL. Øzhayat & Gotfredsen [17] used the full OHIP-49 questionnaire to assess the effects of permanent and removable rehabilitation on OHRQoL, along with other objective dental variables such as aesthetics and chewing abilities. Unlike other studies, they also examined the effects of primary or pathologic deficiencies on patients’ QoL before the onset of rehabilitation, and determined that age, gender, and location of missing teeth did affect the measures. It was found that older age, women, and missing teeth in the aesthetic area exhibited greater aggravation in pre-treatment OHRQoL [17]. Their results showed improvement in both rehabilitation groups, but indicated that the improvement was higher in the removable rehabilitation group. It was noted that rehabilitation treatments that consisted of only replacing molar teeth not only did not cause significant improvement, but also caused new problems that were not reported by patients before treatment [17].

In the study of Swelem et al. [19], an independent analysis of the pre-treatment questionnaires was performed, indicating that the psychological discomfort index had the most significant negative impact on OHRQoL, while the “Functional Limitation” had the most negligible effect. At the end of treatment, all types of treatment exhibited a marked improvement in OHRQoL, but for the same type of treatment, different patients (with age dependence and lack of character) exhibited different effects on oral QoL. Specifically, patients who received fixed rehabilitation showed a slightly greater improvement in QoL compared to young patients who received removable dentures which showed the least improvement.

A meta-synthesis was performed on all articles found as part of the systematic review that had met all required criteria. Seven of the nine studies selected the abbreviated questionnaire (OHIP-14) and only two articles published their research based on the complete questionnaire (OHIP-49). Only one study out of the nine added to the source questionnaires another questionnaire that examined patient satisfaction with treatment [18]. In each study, different results were obtained with respect to the indices whose impact on change in QoL was most significant [14,18,19]. Furthermore—, in addition to the abovementioned indices, each study chose to include several additional parameters, and examined the relationship between them and the degree of improvement in OHRQoL at the end of treatment, including initial lack of sleep [17], aesthetics [17,18,27], chewing abilities [16,18,27], and prosthetic position and retention method [25].

The impact of each of the QoL metrics, as well as the additional clinical parameters examined and their effect on the change in OHRQoL at the end of rehabilitation, along with other information clarified by the results of the study, may help the physician to choose the type of rehabilitation that will produce the best result [27].

Because of the variability of results in each of the reviewed studies, it can be concluded that in this context there is also room for modification of the questionnaires, which will lead to greater and unambiguous clarity on important criteria and metrics.

Despite the differences in the aims of the studies, the types of rehabilitation and the additional variables examined, most of the articles we reviewed (66%) reached the same central conclusion: all patients demonstrated higher post-treatment satisfaction and OHRQoL [14,15,16,17,18,19]. Still, the two questionnaires currently in use nowadays are not routinely used by clinicians in daily practice. Furthermore, the questionnaires miss a niche of central importance in the rehabilitation process, since they do not relate to the role and influence of the attending physician on the success of the treatment [1,20]. Few studies that chose to examine this variable had to add additional questions to the original question that specifically examined the topic [18] reliably. Based on our literature review and meta-synthesis, we constructed a novel questionnaire, which was found to reliable in the current prospective pilot study. We conducted a preliminary study using it to evaluate the effect on the OHRQoL of patients with complete or partial tooth loss. We examined several of restorative dental treatments, which were divided into two categories: fixed or removable rehabilitations.

The new questionnaire contained 31 questions, divided into six pre-defined sub-groups: ‘functional disability’, ‘physiological pain’, ‘psychological discomfort’, ‘physiological disability’, ‘psychological disability’, and ‘social disability’, and achieved satisfactory results.

Our study indicates that dental rehabilitation has a positive effect on OHRQoL across all metrics tested. Patients required to undergo removable rehabilitation treatment exhibited a lower initial status and, therefore, greater improvement at the end of the rehabilitation.

In our attempt to identify different factors that may affect the QoL change (age, gender, and/or duration of rehabilitation) in relation to dental rehabilitation, we found that neither gender nor duration of treatment was found to improve OHRQoL before and after treatment. We did observe a significant relationship between gender and duration of treatment, and the degree of satisfaction with the physician (male patients whose duration of treatment was shorter exhibited greater satisfaction with the physician at the end of rehabilitation). However, in examining whether there was a relationship between the patient’s satisfaction with the physician and the change in quality of life indicators related to oral health, we did not find a significant correlation. The results of our study are in line with findings of other studies, especially those reviewed in our work. Bramanti et al. [14] also found that a positive effect was found in all dimensions of QOL and that the most significant change was observed in the ‘functional disability’ index.

Limitations to this study include its relatively small size, presumably due to reluctance of patients to participate, and since many of those who attend university clinics suffer from various medical conditions and therefore did not meet the inclusion criteria. In order to better assess its suitability for widespread use by dental practitioners, future studies should be conducted on larger sample sizes. It may also opt to explore additional factors, such as the correlation between material improvement in the appearance of teeth at the end of rehabilitation and the ability to make social connections, or to construct a psychological profile for the patient (pre-questionnaire) at the beginning of the treatment and adjusting the type of rehabilitation that will be most appropriate for him.

## 5. Conclusions

This study demonstrated the importance of a novel questionnaire developed to assess psychosocial aspects of dental prosthetic treatments. It was found to be reliable and convenient to use as part of routine dental care. It also allowed for accurate assessment of the psychosocial conditions of patients prior to dental treatments initiation, thus enabling awareness of the dental practitioner towards the patients’ needs, and lead to a positive dental treatment outcome.

## Figures and Tables

**Table 1:** Questionnaire domains and corresponding items.

Domain	Functional Disability	Physiological Pain	Psychological Discomfort	Physiological Disability	Psychological Disability	Social Disability
Items number	Items #1–6	Items #7–10	Items #11–16	Items #17–19	Items #20–24	Items #25–31

**Table 2:** Questionnaire domains internal consistency and reliability [Intraclass correlation coefficient (ICC)] results.

Domain	Functional Disability	Physiological Pain	Psychological Discomfort	Physiological Disability	Psychological Disability	Social Disability
Cronbach’s alpha	α = 0.67	α = 0.77	α = 0.76	α = 0.7	α = 0.7	α = 0.868
ICC (95% confidence interval; *p*)	0.56 (0.31–0.76; <0.001)	0.71 (0.5–0.85; <0.001)	0.69 (0.49–0.84; <0.001)	0.67 (0.42–0.83; <0.001)	0.67 (0.45–0.83; <0.001)	0.84 (0.73–0.92; <0.001)

## Appendix A

**Prosthetic Treatment Questionnaire**

**** Author’s Note**: The section headings were not specified in the version the study participants received.

**Age:**

**Sex:** male/female

**Why I came to treatment:**

- One tooth missing
- I am missing a few teeth
- I am missing missing all teeth
- Replacement of defective permanent restoration
- Repairing a tooth’s aesthetic defect.

**The type of rehabilitation I will receive:**

- Crown
- Front plating
- Bridge
- Crown supported implant
- Bridge supported implant
- Whole denture supported by implants
- Removable whole denture
- Removable partial denture
- Implant supported partial dentures/unknown.

The following questions deal with the feelings and thoughts you have experienced in the past. Each question will ask you how often you felt or thought in a certain way. Although the questions look similar, there are differences between them, so you should treat each question separately.

For each question, select one (and only one) of the following answers:

Never/rarely/sometimes/often/almost always
Unknown/irrelevant

*** Functional disability**

1. Do you have trouble chewing foods of any kind due to problems with your teeth, mouth, or dentures?

Never/rarely/sometimes/often/almost always
Unknown/irrelevant

2. Do you have difficulty pronouncing any words due to problems with your teeth, mouth, or dentures that you wear?

Never/rarely/sometimes/often/almost always
Unknown/irrelevant

3. Do you feel that your sense of taste is impaired by problems with your teeth, mouth, or dentures that you wear?

Never/rarely/sometimes/often/almost always
Unknown/irrelevant

4. Do you feel that the crown/bridge/denture in your mouth does not fit or is not placed properly?

Never/rarely/sometimes/often/almost always
Unknown/irrelevant

5. Have you noticed a tooth in your mouth that doesn’t look good?

Never/rarely/sometimes/often/almost always
Unknown/irrelevant

6. Do you feel that your appearance has been impaired due to problems with your teeth, mouth, or dentures that you wear?

Never/rarely/sometimes/often/almost always
Unknown/irrelevant

*** Physiological pain**

7. Do you feel any pain in your mouth?

Never/rarely/sometimes/often/almost always
Unknown/irrelevant

8. Do you have toothaches?

Never/rarely/sometimes/often/almost always
Unknown/irrelevant

9. Have you had any headaches due to problems with your teeth, mouth, or dentures that you wear?

Never/rarely/sometimes/often/almost always
Unknown/irrelevant

10. Have you avoided eating certain foods due to the pain that comes from your teeth, mouth, or dentures that you wear?

Never/rarely/sometimes/often/almost always
Unknown/irrelevant

*** Psychological discomfort**

11. Are you concerned about dental problems?

Never/rarely/sometimes/often/almost always
Unknown/irrelevant

12. Have dental problems made you miserable?

Never/rarely/sometimes/often/almost always
Unknown/irrelevant

13. Do you feel uncomfortable with your appearance due to problems that arise from your teeth, mouth, or dentures that you wear?

Never/rarely/sometimes/often/almost always
Unknown/irrelevant

14. Have you been in stress due to problems that arise from your teeth, mouth, or dentures that you wear?

Never/rarely/sometimes/often/almost always
Unknown/irrelevant

15. Do you feel dissatisfied with the look of your teeth when you look in the mirror?

Never/rarely/sometimes/often/almost always
Unknown/irrelevant

16. Do you feel jealous of people who have prettier teeth than yours?

Never/rarely/sometimes/often/almost always
Unknown/irrelevant

*** Physiological disability**

17. Have you felt that your overall health has been damaged by problems that arise from your teeth, mouth, or dentures that you wear?

Never/rarely/sometimes/often/almost always
Unknown/irrelevant

18. Do you feel that you are having trouble fulfilling your regular work due to problems that arise from your teeth, mouth, or dentures that you wear?

Never/rarely/sometimes/often/almost always
Unknown/irrelevant

19. Do you have difficulty functioning due to problems that arise from your teeth, mouth, or dentures that you wear?

Never/rarely/sometimes/often/almost always
Unknown/irrelevant

*** Psychological disability psychological disability**

20. Have you been moody due to problems with your teeth, mouth, or dentures that you wear?

Never/rarely/sometimes/often/almost always
Unknown/irrelevant

21. Do you feel that you are placed under a lot of stress due to problems with your teeth, mouth, or dentures that you wear?

Never/rarely/sometimes/often/almost always
Unknown/irrelevant

22. Have you been depressed due to problems that arise from your teeth, mouth, or dentures that you wear?

Never/rarely/sometimes/often/almost always
Unknown/irrelevant

23. Have you been embarrassed by problems that arise from your teeth, mouth, or dentures you wear?

Never/rarely/sometimes/often/almost always
Unknown/irrelevant

24. Have you been anxious about problems that arise from your teeth, mouth, or dentures you wear?

Never/rarely/sometimes/often/almost always
Unknown/irrelevant

*** Social disability**

25. Do you avoid going out for leisure because of problems that arise from your teeth, mouth, or dentures that you wear?

Never/rarely/sometimes/often/almost always
Unknown/irrelevant

26. Do you feel that you have trouble making new social connections due to problems with your teeth, mouth, or dentures that you wear?

Never/rarely/sometimes/often/almost always
Unknown/irrelevant

27. Have you generally felt that life is less satisfying because of problems that arise from your teeth, mouth, or dentures that you wear?

Never/rarely/sometimes/often/almost always
Unknown/irrelevant

28. Do you feel that you avoid smiling with your mouth open in an attempt to hide your teeth?

Never/rarely/sometimes/often/almost always
Unknown/irrelevant

29. Do you catch yourself covering your mouth with your hands in an attempt to hide it during social gatherings?

Never/rarely/sometimes/often/almost always
Unknown/irrelevant

30. Do you think or feel that people are staring at your teeth?

Never/rarely/sometimes/often/almost always
Unknown/irrelevant

31. When you meet new people you don’t know well, are you worried about what they will think of you after seeing your teeth?

Never/rarely/sometimes/often/almost always
Unknown/irrelevant

**** Part B appeared only in a questionnaire distributed to study participants for the second time, at the end of rehabilitation.**

*** Importance of the treating physician**

1. Have you been worried about going to the dentist?

Never/rarely/sometimes/often/almost always
Unknown/irrelevant

2. Did you feel, at any time of the treatment, that the dentist did not properly explain to you the treatment you were going to go through or that they didn’t explain you the different options you were facing?

Never/rarely/sometimes/often/almost always
Unknown/irrelevant

3. Did you think or feel that there were things you did not receive from your dentist?

Never/rarely/sometimes/often/almost always
Unknown/irrelevant

4. Did you think or feel that the treatment you received was good?

Never/rarely/sometimes/often/almost always
Unknown/irrelevant

5. Did you think or feel that your dentist was impatient with you during the process?

Never/rarely/sometimes/often/almost always
Unknown/irrelevant

6. Did you think or feel that the attending physician gave you a personal treatment plan?

Never/rarely/sometimes/often/almost always
Unknown/irrelevant

* The answers to questions 4 and 6 were reversed in the statistical analysis to fit the research conditions (so that a high-value answer characterized dissatisfaction)

## Appendix B

**All Psychosocial Impact of Dental Aesthetics Questionnaire (PIDAQ) entries**

***Dental Self-Confidence***

- I am proud of my teeth.
- I like to show my teeth when I smile.
- I am pleased when I see my teeth in the mirror.
- My teeth are attractive to others.
- I am satisfied with the appearance of my teeth.
- I find my tooth position to be very nice.

***Social Impact***

- I hold myself back when I smile so my teeth don’t show so much.
- If I don’t know people well I am sometimes concerned what they might think about my teeth.
- I’m afraid other people could make offensive remarks about my teeth.
- I am somewhat inhibited in social contacts because of my teeth.
- I sometimes catch myself holding my hand in front of my mouth to hide my teeth.
- Sometimes I think people are staring at my teeth.
- Remarks about my teeth irritate me even when they are meant jokingly.
- I sometimes worry about what members of the opposite sex think about my teeth.

***Psychological Impact***

- I envy the nice teeth of other people.
- I am somewhat distressed when I see other people’s teeth.
- Sometimes I am somewhat unhappy about the appearance of my teeth.
- I think most people I know have nicer teeth than I do.
- I feel bad when I think about what my teeth look like.
- I wish my teeth looked better.

***Aesthetic Concern***

- I don’t like to see my teeth in the mirror.
- I don’t like to see my teeth in photographs.
- I don’t like to see my teeth when I look at a video of myself.

## Appendix C

**Table A1:** Oral health impact profile (OHIP 14).

OHIP-14					
In the Last Six Months	Never	Hardly ever	Occasionally	Fairly Often	Very Often
(1) Have you had trouble pronouncing any words because of problem with your teeth, mouth or dentures?					
(2) Have you felt that your sense of taste has worsened because of problems with your teeth, mouth or dentures?					
(3) Have you had painful aching in your mouth?					
(4) Have you found it uncomfortable to eat any foods because of problems with your teeth, mouth or dentures?					
(5) Have you been worried by dental problems?					
(6) Have you felt tense because of problems with teeth, mouth or dentures?					
(7) Has your diet been unsatisfactory because of problems with teeth, mouth or dentures?					
(8) Have you had to interrupt meals because of problems with teeth, mouth or dentures?					
(9) Have you found it difficult to relax because of problems with teeth, mouth or dentures?					
(10) Have you been a bit embarrassed because of problems with teeth, mouth or dentures?					
(11) Have you been a bit irritable with other people because of problems with teeth, mouth or dentures?					
(12) Have you had difficulty doing you usual jobs because of problems with teeth, mouth or dentures?					
(13) Have you felt that life in general was less satisfying because of problems with teeth, mouth or dentures?					
(14) Have you been totally unable to function because of problems with teeth, mouth or dentures?					
